# Supplementary figures and images for: Identification of the NAC Transcription Factor Family during Early Seed Development in Akebia trifoliata (Thunb.) Koidz
Source: Plants (Basel). 2023 Mar 31;12(7):1518. doi: 10.3390/plants12071518 (PMC10096588; doi:10.3390/plants12071518)

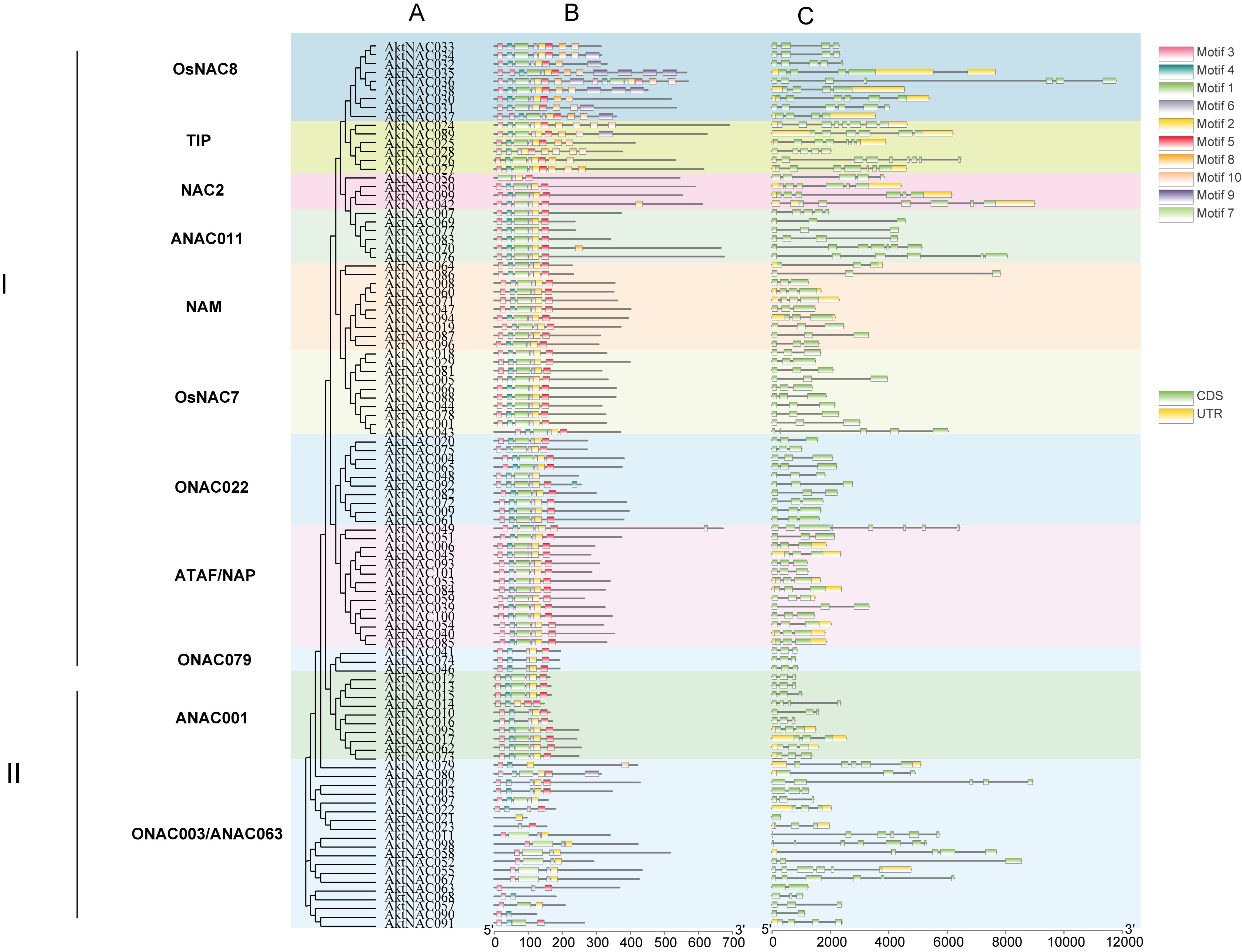

Supplement: Supplementary file 1 [file plants-12-01518-s001.zip › Supplementary Figure S1.tif]

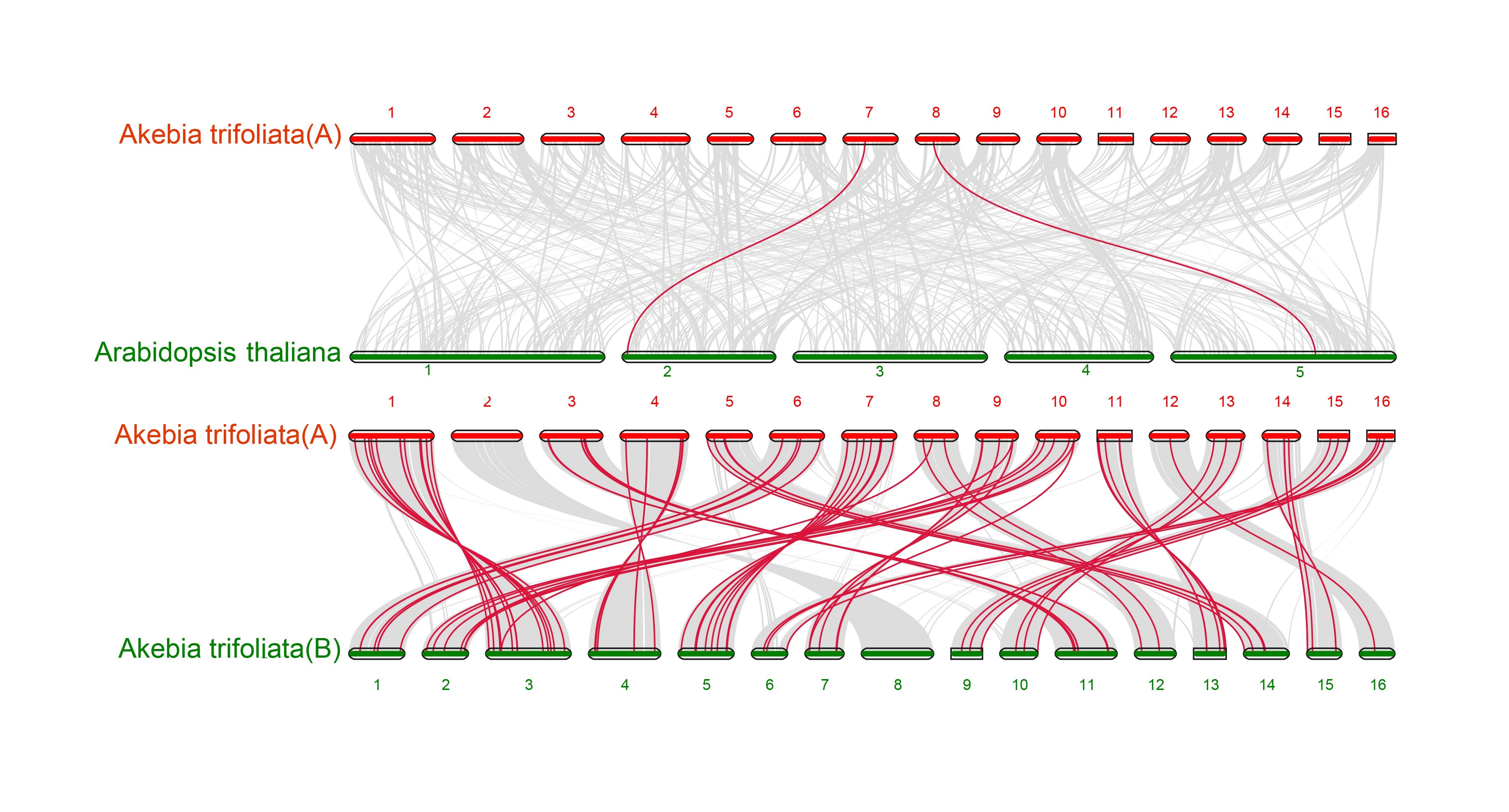

Supplement: Supplementary file 1 [file plants-12-01518-s001.zip › Supplementary Figure S3.jpg]

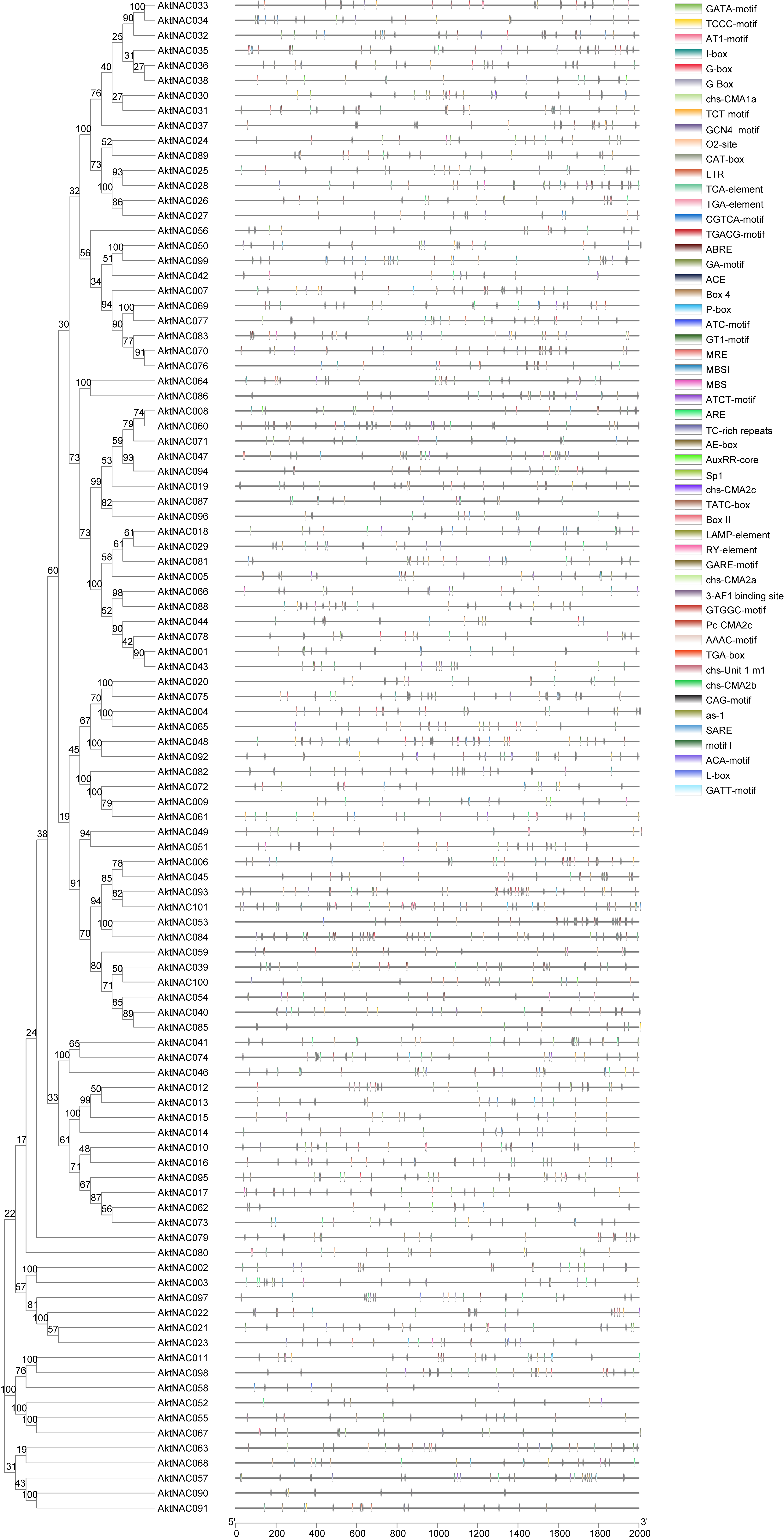

Supplement: Supplementary file 1 [file plants-12-01518-s001.zip › Supplementary Figure S4.tif]

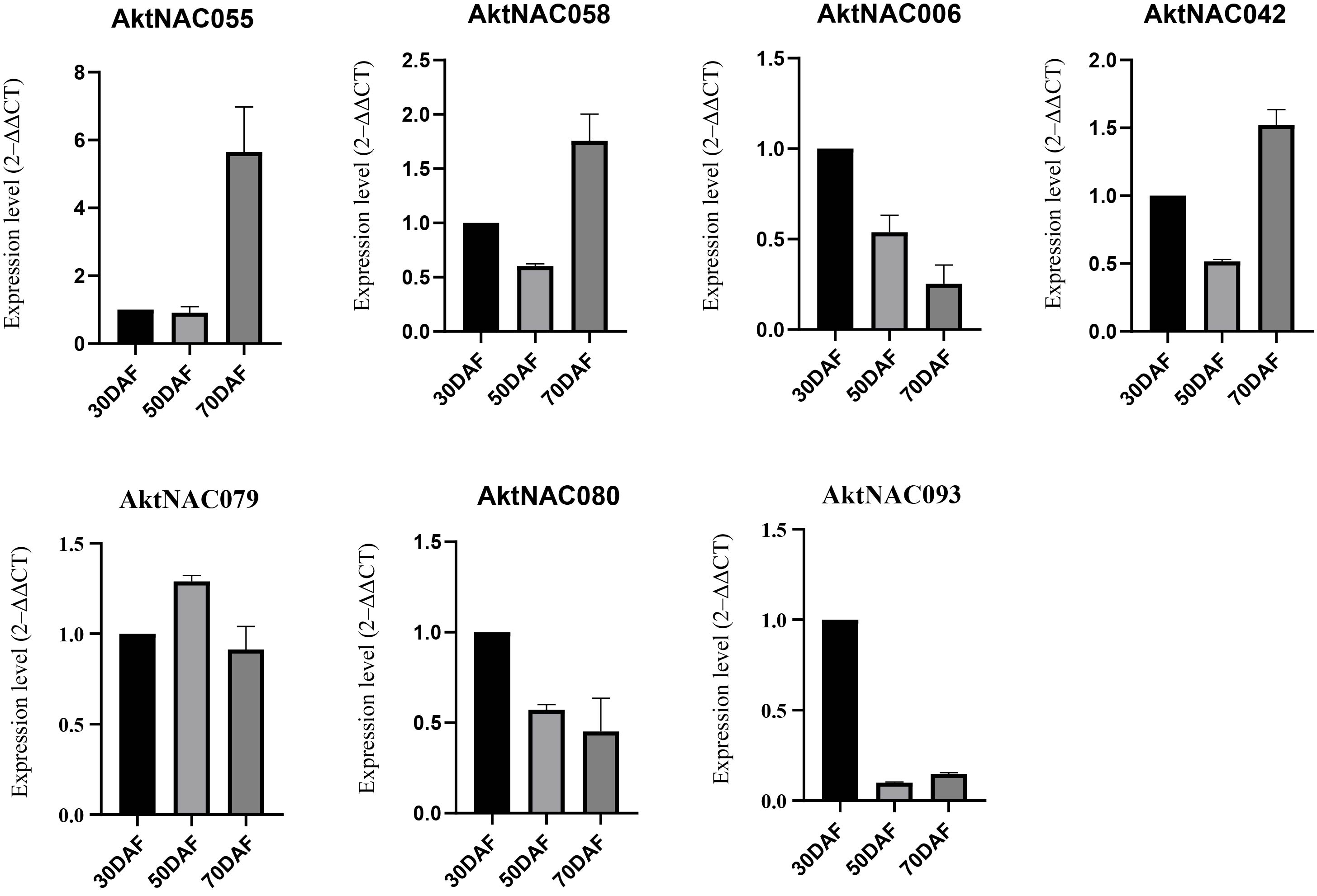

Supplement: Supplementary file 1 [file plants-12-01518-s001.zip › Supplementary Figure S5.jpg]
